# Surface expression of protein A on magnetosomes and capture of pathogenic bacteria by magnetosome/antibody complexes

**DOI:** 10.3389/fmicb.2014.00136

**Published:** 2014-04-03

**Authors:** Jun Xu, Junying Hu, Lingzi Liu, Li Li, Xu Wang, Huiyuan Zhang, Wei Jiang, Jiesheng Tian, Ying Li, Jilun Li

**Affiliations:** ^1^Department of Microbiology, College of Biological Sciences, China Agricultural UniversityBeijing, China; ^2^Food Safety Testing Centre, Beijing Entry-Exit Inspection and Quarantine BureauBeijing, China

**Keywords:** *Magnetospirillum gryphiswaldense*, magnetosome, MamF, surface display, protein A, functionalization, *Vibrio parahaemolyticus*

## Abstract

Magnetosomes are membrane-enclosed magnetite nanocrystals synthesized by magnetotactic bacteria (MTB). They display chemical purity, narrow size ranges, and species-specific crystal morphologies. Specific transmembrane proteins are sorted to the magnetosome membrane (MM). MamC is the most abundant MM protein of *Magnetospirillum gryphiswaldense* strain MSR-1. MamF is the second most abundant MM protein of MSR-1 and forms stable oligomers. We expressed staphylococcal protein A (SPA), an immunoglobulin-binding protein from the cell wall of *Staphylococcus aureus*, on MSR-1 magnetosomes by fusion with MamC or MamF. The resulting recombinant magnetosomes were capable of self-assembly with the Fc region of mammalian antibodies (Abs) and were therefore useful for functionalization of magnetosomes. Recombinant plasmids pBBR-mamC-spa and pBBR-mamF-spa were constructed by fusing *spa* (the gene that encodes SPA) with *mamC* and *mamF*, respectively. Recombinant magnetosomes with surface expression of SPA were generated by introduction of these fusion genes into wild-type MSR-1 or a *mamF* mutant strain. Studies with a Zeta Potential Analyzer showed that the recombinant magnetosomes had hydrated radii significantly smaller than those of WT magnetosomes and zeta potentials less than −30 mV, indicating that the magnetosome colloids were relatively stable. Observed conjugation efficiencies were as high as 71.24 μg Ab per mg recombinant magnetosomes, and the conjugated Abs retained most of their activity. Numbers of *Vibrio parahaemolyticus* (a common pathogenic bacterium in seafood) captured by recombinant magnetosome/Ab complexes were measured by real-time fluorescence-based quantitative PCR. One mg of complex was capable of capturing as many as 1.74 × 10^7^ *Vibrio* cells. The surface expression system described here will be useful for design of functionalized magnetosomes from MSR-1 and other MTB.

## Introduction

The bacterium *Vibrio parahaemolyticus* is a major cause of food-borne illnesses resulting from consumption of raw seafood and is involved in gastroenteritis, wound infection, and septicemia (Newton et al., 2012). Conventional methods for the detection of *V. parahaemolyticus* include the use of selective, differential agar media, biochemical testing, and examination of colony morphology (Kaysner and DePaola, 2004). Such methods usually involve time-consuming laboratory procedures and provide limited knowledge regarding pathogenicity.

Techniques based on polymerase chain reaction (PCR) have been used increasingly in recent years to detect pathogenic strains of *V. parahaemolyticus* by targeting the amplification of specific gene sequences with appropriate primers. A thermolabile direct hemolysin (TLH) is specific for *V*. *parahaemolyticus*. Its gene, *tlh*, is a frequently used target in various detection strategies (Su and Liu, 2007). However, PCR in this case is inhibited by a variety of substances present in food or in the environment (Rossen et al., 1992; Powell et al., 1994; Waleed and Peter, 2000). Removal of such inhibitory substances is a crucial step in the preparation of template DNA samples for PCR-based detection of food pathogens.

Immunomagnetic separation (IMS) is a powerful technique for the specific isolation and concentration of target bacteria from food samples (Spanová et al., 2003; Ångela et al., 2008; Mercanoglu et al., 2009). Magnetosomes (also termed bacterial magnetic particles, or BMPs; this abbreviation is used hereafter for convenience) are being used increasingly as carriers for IMS assays (Arakaki et al., 2008; Faivre and Schüler, 2008). BMPs are synthesized by MTB and are composed of membrane-enclosed, single-domain ferrimagnetic iron oxide (magnetite, Fe_3_O_4_), or iron sulfide (greigite, Fe_3_S_4_) crystals (Schüler, 2002). At least 20 proteins have been identified on the magnetosome membrane (MM) of *Magnetospirillum gryphiswaldense* strain MSR-1 (hereafter termed “MSR-1”). Grünberg et al. (2004) reported that MamC was the most abundant MM-associated protein and that MamF was the second most abundant and the most stable. Expression of foreign functional proteins on the BMP surface can be facilitated by genetic engineering of MM-associated proteins. Many recent studies have attempted to produce various types of functionalized BMPs, for instance by the BMP-specific display of functional moieties, such as enzymes, coupling groups, gold particles, or oligonucleotides (BMP surface display system, Yoshino et al., 2010).

In the present study, staphylococcal protein A (SPA) was expressed on magnetosomes by fusion with MamC or MamF. SPA is an immunoglobulin G-binding protein (antibody-binding protein) encoded by the *spa* gene and can be isolated from the cell wall of *Staphylococcus aureus*. It binds the heavy chain within the fragment crystallizable region (Fc region, or tail region) of most immunoglobulins under a wide variety of conditions (Sidorin and Solov'eva, 2011). The resulting recombinant magnetosomes (BMP-A) were capable of self-assembly with many mammalian antibodies (Abs) without a loss of Ab activity. These recombinant BMPs were characterized and their Ab-binding efficiencies were evaluated. The capture efficiencies of magnetosome complexes (BMP-A-Ab) for *V*. *parahaemolyticus* were also investigated.

## Materials and methods

### Bacterial strains, primers, probes, culture media, and growth conditions

The bacterial strains, mutants, plasmids, and primers used in this study are listed in Tables 1, 2. *Escherichia coli* (*E. coli*) strains were grown at 37°C in Luria-Bertani (LB) medium (Sambrook and Russel, 2001). *M. gryphiswaldense* strains were grown at 30°C in sodium lactate/ ammonium chloride/yeast extract (L AY) medium as described previously (Liu et al., 2008). Heat-killed cells of *V. parahaemolyticus* strain 09vp109 were from the Beijing Entry-Exit Inspection and Quarantine Bureau (Beijing, China).

**Table 1:** ***M*. *gryphiswaldense* strains and plasmids in this study**.

	**Strain or plasmid**	**Genotype**	**Source**
**Strains**	*M. gryphiswaldense* MSR-1	Wild-type (WT)	DSMZ
	*M. gryphiswaldense* ΔF	MSR-1 mamF mutant	Present study
	*M. gryphiswaldense* MSR-CA	MSR-1 harboring pBBR-mamC-spa; Nx^r^, Km^r^	Present study
	*M. gryphiswaldense* MSR-FA	MSR-1 harboring pBBR-mamF-spa; Nx^r^, Km^r^	Present study
	*M. gryphiswaldense* ΔF-FA	mamF mutant harboring pBBR-mamF-spa; Nx^r^, Km^r^, Gm^r^	Present study
	*S. aureus* ATCC 6538	WT	CGMCC
	*E*. *coli* DH5α	*endA1 hsdR17[r^−^m^+^]supE44 thi-1 recA1 gyrA[NalR]RelA relA1*Δ*[lacZYA-argF]U169 deoR[ø80]Δ (M15)*]	Stock culture in our laboratory
	*E. coli* S17-1	*Pro thi hsdR recA*, chromosomal integration of RP4-2-Tc::Mu-Km::Tn7, Sm^r^Tra^+^	
	*V. parahaemolyticus* 09vp109	WT	
**Plasmids**	pUC-GM	Amp^r^, pUC18 harboring gentamicin resistance gene	Laboratory collection
	pUX19	Suicide vector; Km^r^	
	PMD18-T simple	PCR cloning vector; Amp^r^	TaKaRa
	pBBR1MCS-2	Expression vector/LacZ promoter; Km^r^	Kovach et al., 1995
	pBBR-mamC-spa	pBBR1MCS-2 harboring gene fragment of mamC-spa; Km^r^	Present study
	pBBR-mamF-spa	pBBR1MCS-2 harboring gene fragment of mamF-spa; Km^r^	Present study

*DSMZ, German Collection of Microorganisms and Cell Cultures GmbH; CGMCC, China General Microbiological Culture Collection Center*.

**Table 2:** **Primers used for PCR**.

**Target gene**	**Primer**	**Oligonucleotide sequences(s) (5′ to 3′)**
*mamC*	F-mamC^e^	^a^*CCGGAATTC*GCCTGACCCTTGAATTAAGGAC
	R-mamC^f^	^b^*GGAACCGCCGCCACCAGAGCCACCACCGCCGGA*GGCCAATTCTTCCCTCAGAATG
*mamF*	F-mamF^e^	^a^*CCGGAATTC*GCGAGGGCAAAGCAATGG
	R-mamF^f^	^b^*GGAACCGCCGCCACCAGAGCCACCACCGCCGGA*GATCAGGGCGACTACATGG
*spa*	F-spa^e^	^c^*TCTGGTGGCGGCGGTTCCGGTGGCGGTGGC*AAAAAGAAAAACATTTATTCAATTCGTAAACTA
	R-spa^f^	^d^*CGCGGATCC*TTATAGTTCGCGACGACGTCC
*mamF* upstream fragment	mamF-D1	CGGGGTACCCTGATGGGAAAGACCGTGCT
	mamF-D2	AACTGCAGAGATAACAACAACCAACGCCC
*mamF* downstream fragment	mamF-G1	GCTCTAGACGACTTCTTCATCGCTCTGTG
	mamF-G2	CGGGGTACCCATTGCTTTGCCCTCGCTT
*tlh*	F-*tlh*^e^	TGTTCGAGACGCTAACTTCTG
	R-*tlh*^f^	AAACTTCTCAGCACCAGACG

a recognition site of restriction endonuclease BamHI;

b Linker sequence;

c complementary linker sequence of sequence b;

d recognition site of restriction endonuclease EcoRI;

e forward primer;

f *reverse primer*.

Bicinchoninic acid (BCA) kits were from Pierce Biotechnology (Rockford, Illinois, USA). Abs were prepared by Kirkegaard and Perry Laboratories (KPL) Biotechnology (Gaithersburg, Maryland, USA). Other chemical reagents were from Beijing Chemical Reagents Co., China.

Primers and probes were designed using ABI 7000 Primer Express software (http://www.lifetechnologies.com/global/en/home/technical-resources/software-downloads/abi-prism-7000-sequence-detection-system.html). The probe used for real-time fluorescence quantitative PCR (FQ-PCR) was labeled with 6-carboxyfluorescein reporter dye (FAM) at the 5′-end and 6-carboxy-tetramethylrhodamine quencher dye (TAMRA) at the 3′-end.

### Construction of recombinant plasmids and strains

Mutant strain *M. gryphiswaldense* ΔF was constructed by replacing *mamF* with the gentamicin resistance gene (aminoglycoside acetyltransferase gene, *aac*). Plasmid pUC-GM was digested by *Kpn*I to generate an *aac* gene fragment. The upstream and downstream fragments of *mamF* gene were amplified by the corresponding primers (Table 2) from genomic DNA of MSR-1, and referred to as U and D, respectively. Fragments U and D were digested by *Xba*I/*Kpn*I and *Kpn*I/*Pst*I and then connected with the *aac* gene fragment by T4 DNA ligase to generate a U-aac-D fragment. The U-aac-D fragment was cloned into a suicide plasmid pUX19. The recombinant plasmid was transformed into *E. coli* S17-1 and then transferred into MSR-1 by biparental conjugation. Mutant bacterial strains were screened as described previously (Liu et al., 2008).

**Figure 1:**
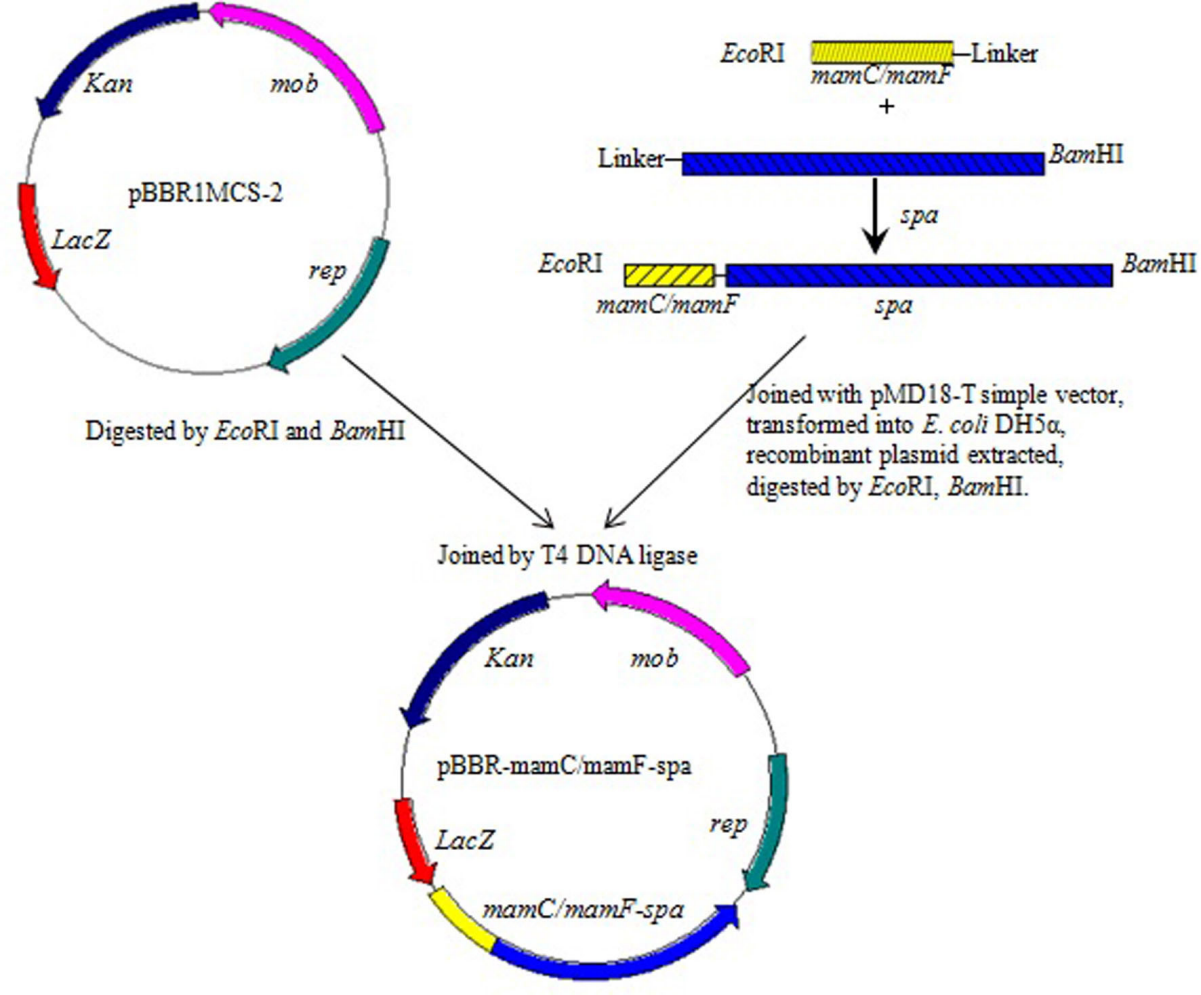
**Construction of plasmids pBBR-mamC-spa and pBBR-mamF-spa**.

*mamC*, *mamF*, and *spa* genes were amplified from genomic DNA of MSR-1 or *S. aureus* ATCC 6538 by the corresponding primers (Table 2). The start codon of *spa* and the stop codons of *mamC* and *mamF* were removed during amplification. The three above fragments were recovered to generate *mamC-spa* and *mamF-spa* fragments by fusion PCR (Komeili et al., 2004). These two fragments were respectively cloned into pMD18-T simple cloning vector and transformed into *E. coli* DH5α. After overnight culture of the recombinant strains, two plasmids were extracted and digested with *Eco*RI and *Bam*HI. The recovered fragments were then cloned into the broad host plasmid vector pBBR1MCS-2, resulting in plasmids pBBR-mamC-spa and pBBR-mamF-spa, respectively (Figure 1). These plasmids contained a linker consisting of 15 amino acids between the *spa* and *mam* genes, and recognition sites of restriction endonucleases *Bam*HI and *Eco*RI flanking the sides of the *mam-spa* fusion genes.

Plasmids pBBR-mamC-spa and pBBR-mamF-spa were introduced into *E. coli* S17-1 by transformation (Sambrook and Russel, 2001) and then transferred into MSR-1 or *M*. *gryphiswaldense* ΔF by conjugation as described previously (Liu et al., 2008).

### Preparation of magnetosome-Ab complexes

Magnetosomes or recombinant magnetosomes (BMP-As) of *M. gryphiswaldense* strains were isolated and purified as described previously (Li et al., 2010). The membrane proteins of BMPs or BMP-As were extracted as described by Grünberg et al. (2004) and identified by Northern blotting. The proteins of each sample (generally from 0.25 mg magnetosomes) were separated by SDS-PAGE, and the bands were transferred onto a nitrocellulose membrane by electroblotting and blocked overnight at 4°C. A dilute solution of primary Ab (mouse mAb, 0.5–5.0 mg/mL) was incubated with the membrane under gentle agitation for 1 h at room temperature. The membrane was rinsed to remove unbound primary Ab and then placed in a solution of secondary Ab (goat anti-mouse IgG) for 1 h. The secondary Ab was linked to alkaline phosphatase, which was then used to catalyze 5-bromo-4-chloro-3-indolyl phosphate (BCIP) and nitro blue tetrazolium (NBT) into a blue precipitate in proportion to the amount of protein.

The hydrated radii and zeta potentials of magnetosomes were analyzed by a Zeta Potential Analyzer (Brookhaven Instruments Corp., Long Island, State of New York USA). Samples were prepared as described by Takahashi et al. (2010).

Chemical modification of magnetosomes by bis(sulfosuccinimidyl) suberate (BS^3^) was performed as described previously (Li et al., 2010). The procedure for self-assembly of recombinant magnetosome was as follows. BMP-A or Ab (each 1 mg) was resuspended in 1 mL of 1 mM phosphate-buffered saline (PBS; pH 7.4). The magnetosome and Ab suspensions were mixed, placed in a mild ultrasonic bath (30 W) for 2 min, and incubated on a rotary shaker at 150 × g for 60–90 min. The resulting BMP-A-Ab complexes were isolated by a NdFeB magnet, washed three times with 1 mL of 10 mM PBS (pH 7.4), and resuspended in 500 μL of 1 mg/mL Ab solution. After repeated dispersion, incubation, collection, and washing as above, the complexes were blocked with sterile 0.5% BSA solution overnight at 4°C and stored.

Ab concentrations before and after reaction with magnetosomes were determined using a BCA kit, and linkage rates were calculated by the equation:

Linkage rate (μg Ab/mg magnetosomes) = (C1–C2) × V/M

where C1 = Ab concentration before reaction with magnetosomes; C2 = Ab concentration after reaction with magnetosomes; V = volume of Ab solution reacting with magnetosomes; M = weight of magnetosomes conjugated with Ab. C1 and C2 were calculated by the equation γ = kx, where γ = OD_562_ detected by the BCA kit; x = Ab concentration; k = slope.

### IMS and capture efficiency detection of *V.parahaemolyticus* by FQ-PCR

Serially 10-fold diluted *Vibrio* suspensions (10^−3^, 10^−4^, 10^−5^, 10^−6^; each 1 mL) were mixed with 1 mg BMP-A-Ab complexes. The mixtures were dispersed by sonication for 2 min and incubated on a rotary shaker (150 × g) for 1 h at room temperature. BMP-A-Ab-*Vibrio* complexes were isolated using a magnet and washed three times with 1 mL of 10 mM PBS buffer to remove free bacterial cells. Control mixtures of *Vibrio* and BMP-As (without Ab) were incubated in PBS under the same conditions. The resulting precipitate was resuspended in 100 μL PBS. The concentrated cell suspension was incubated for 10 min at 98°C and centrifuged at 10,000 × g for 3 min at room temperature. The supernatants were used as templates. FQ-PCR was performed as described previously (Li et al., 2010).

*V*. *parahaemolyticus* strains produce species-specific TLHs, and the *tlh* gene has been used as a probe to confirm the identity of *Vibrio* species (McCarthy et al., 1999). We measured the fluorescence intensity of tlh FQ-PCR products to estimate the number of *Vibrio* cells trapped by BMP-A-Ab complexes. Standard curves were constructed using a 10^−1^–10^−6^ serial 10-fold dilution of *Vibrio* genomic DNA, and the threshold cycle (Ct) value was plotted against the log of DNA weight (ng) by linear regression. The average weight of a pair of DNA bases is 1 × 10^−21^ g, and the whole genome of *V*. *parahaemolyticus* (5165770 bp; Makino et al., 2003) was estimated to weigh 5.2 × 10^−15^ g. The number of *Vibrio* was calculated as the amount of DNA divided by the weight of genome.

## Results

### Construction of recombinant plasmids and strains encoding fusion proteins

SPA is an immunoglobulin G-binding protein. Magnetosome-specific expression of SPA could facilitate efficient localization and appropriate orientation of various Abs on the surface of magnetosomes. In the present study, SPA was expressed on magnetosomes by fusing its gene (*spa*; 1503 bp) with the abundant MM protein genes *mamC* (378 bp) or *mamF* (336 bp) from *M. gryphiswaldense*. The fusion genes *mamC-spa* and *mamF-spa* were generated by fusion PCR from *mamC*, *mamF*, or *spa* PCR products. These genes were cloned into the broad host plasmid vector pBBR1MCS-2 (5144 bp), resulting in plasmids pBBR-mamC-spa and pBBR-mamF-spa, which were then introduced into MSR-1 by conjugation. The recombinant strains harboring plasmid pBBR-mamC-spa and pBBR-mamF-spa were termed MSR-CA and MSR-FA, respectively.

To increase the amount of SPA on the recombinant magnetosomes (BMP-As), a *mamF* mutant strain of *M. gryphiswaldense* was constructed by allelic gene replacement. The native *mamF* gene in the MSR-1 genome was replaced by a gentamicin resistance gene (aminoglycoside acetyltransferase gene; *aac*), resulting in strain ΔF. The mutant strain harboring plasmid pBBR-mamF-spa was termed ΔF-FA. Recombinant magnetosomes from strains MSR-1, MSR-CA, MSR-FA, and ΔF-FA were termed BMP-WT, BMP-CA, BMP-FA, and ΔF-BMP-FA, respectively.

**Figure 2:**
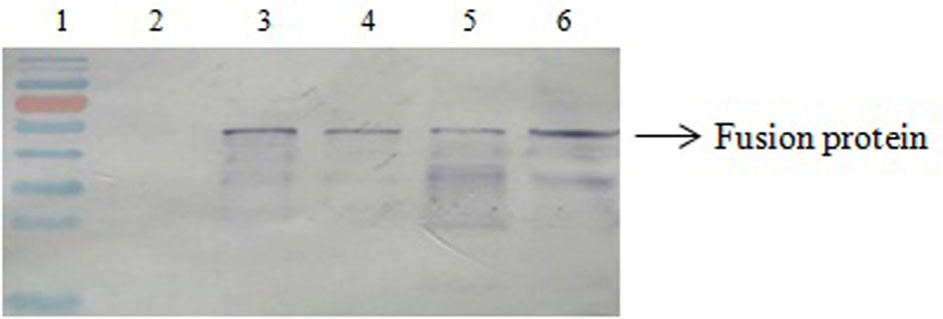
**Western blotting results**. Lane 1, molecular markers; Lane 2, BMP-WT; Lane 3, BMP-CA; Lane4, fusion protein; Lane 5, BMP-FA; Lane 6, ΔF-BMP-FA.

The expression of SPA on magnetosomes was confirmed by western blotting. The membrane proteins of purified magnetosomes were separated by SDS-PAGE and transferred onto a nitrocellulose membrane by electroblotting. The protein bands were colored by sequential treatment with primary Ab, secondary Ab, and BCIP-NBT. SPA was found in all the recombinant magnetosomes (Figure 2).

### Characterization of recombinant magnetosomes

**Figure 3:**
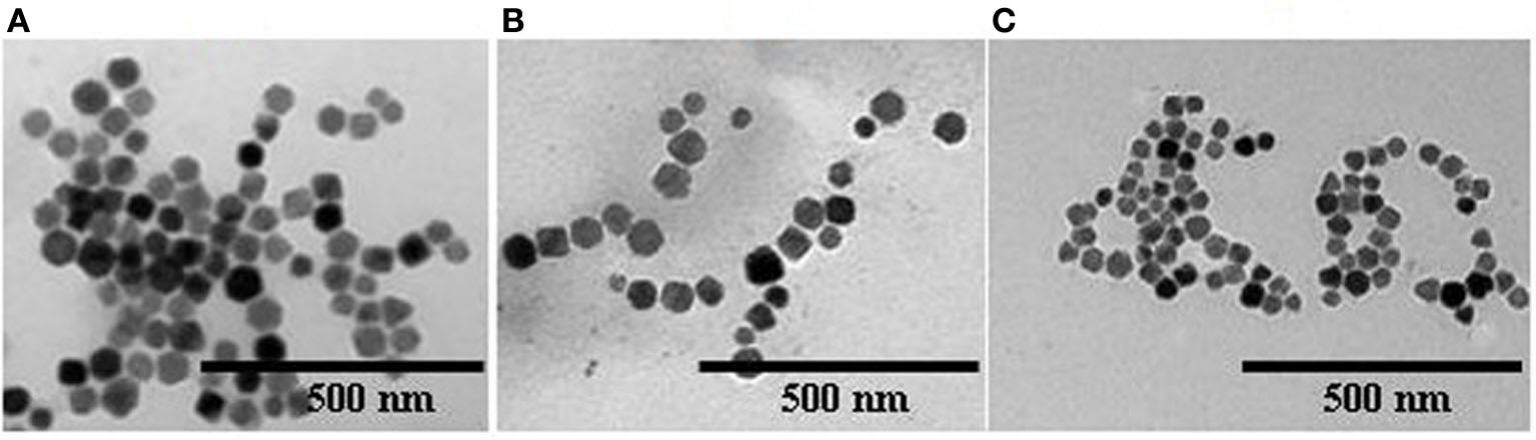
**Electron micrographs of magnetosomes from various strains (A)**, BMP-WT; **(B)**, BMP-FA; **(C)**, ΔF-BMP-FA.

Strains as above were collected by centrifugation after 3 days of culture, and their magnetosomes were isolated and purified. Transmission electronic microscopy (Figure 3) showed generally similar morphology of WT magnetosomes (diameter range 35–84 nm) and recombinant magnetosomes. The magnetosomes of ΔF-FA were slightly smaller (24–48 nm) than those of the other strains.

The hydrated radii and zeta potentials of magnetosomes were analyzed by a Zeta Potential Analyzer. All zeta potentials were lower than −30 mV, indicating that the aqueous colloids were moderately stable. The expression of foreign proteins can affect the amount of electrical charge on interfaces between magnetosomes and aqueous solutions. The large hydrated radii of BMP-WTs indicated that several BMP-WT gathered into a larger particle in suspension. The expression of foreign proteins on magnetosomes in the case of BMP-CA and BMP-FA was associated with smaller values of hydrated radii and polydispersity, indicating improved dispersity and uniformity. ΔF-BMP-FA had the smallest hydrated radii and best dispersity because they had the largest amount of foreign proteins.

### Efficiency of magnetosome linkage to polyclonal Ab

**Figure 4:**
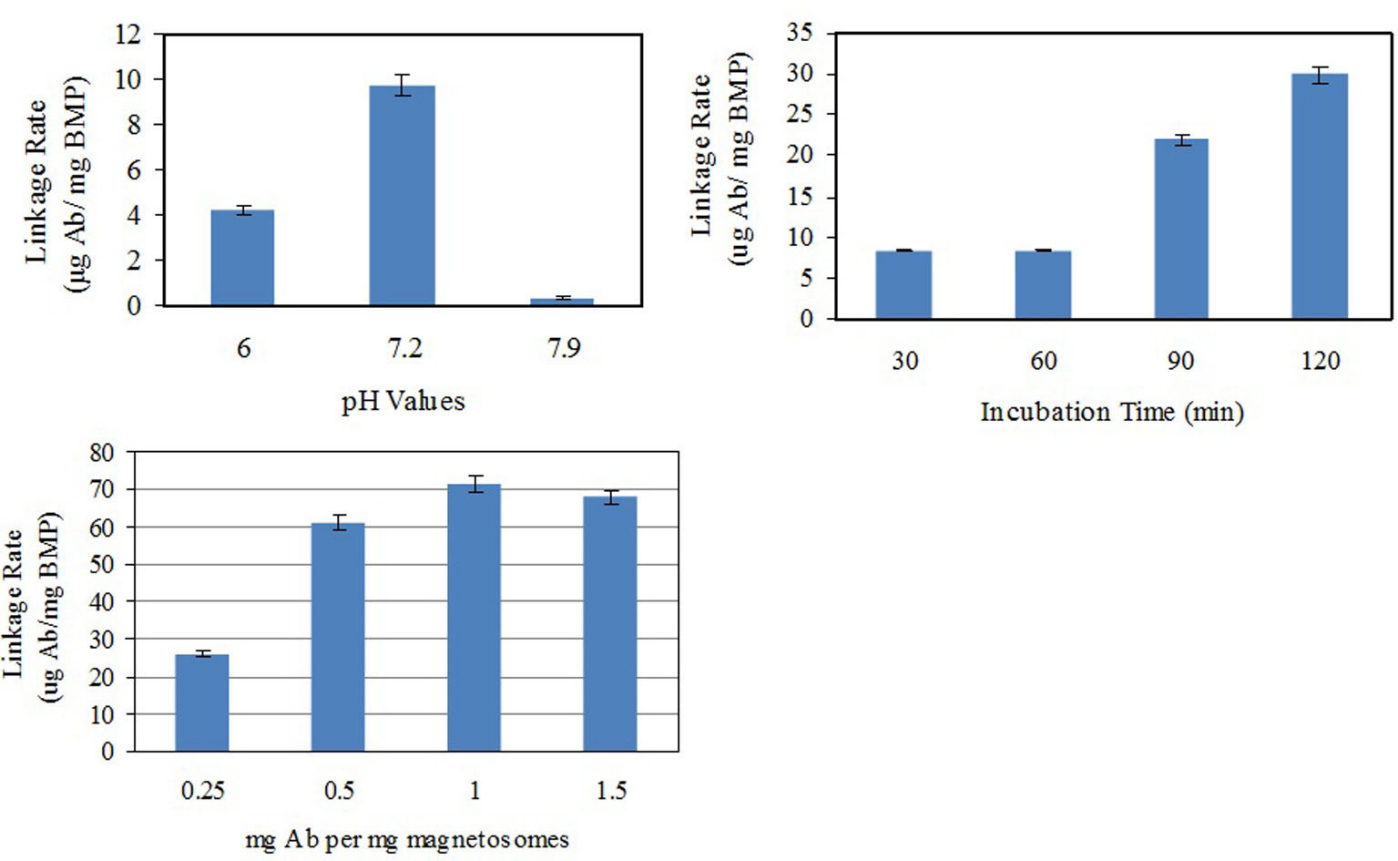
**Optimization of conditions for linkage of BMP-CA to Ab**.

Polyclonal Abs were linked with SPA on the surface of magnetosomes by self-assembly to form functional magnetic carriers (BMP-Ab) for capturing *Vibrio*. In an attempt to improve linkage rate, various values of pH (6.0, 7.2, 7.9), incubation time (30, 60, 90, 120 min), and ratio of Ab to magnetosomes (250 μg, 500 μg, 1.0 mg, and 1.5 mg Ab per mg magnetosomes) were used for the linkage of BMP-CA to Ab (Figure 4). The maximal linkage rate was achieved with pH 7.2, incubation time 120 min, and ratio 1:1.

**Figure 5:**
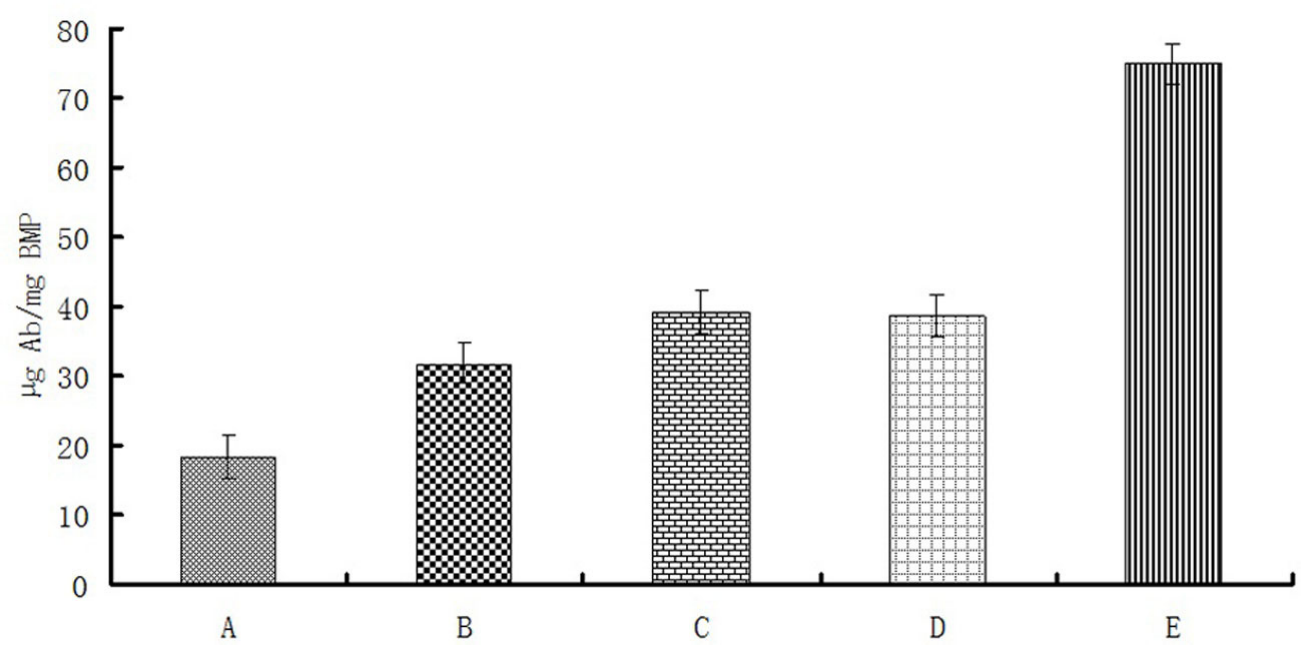
**Magnetosome-Ab linkage rates for different linkage methods under optimal conditions as in Figure 4**. (A), Nonspecific adsorption of BMP-WT on Ab;(B),BS^3^ linkage of BMP-WT to Ab; (C), self-assembly of BMP-CA and Ab; (D), self-assembly of BMP-FA and Ab; (E) self-assembly of ΔF-BMP-FA and Ab.

Linkage rates of different magnetosome-Ab linking methods were compared under these optimal conditions (Figure 5). BMP-WTs were linked to Ab by nonspecific adsorption or BS^3^, and BMP-CA and ΔF-BMP-FA were linked to Ab by self-assembly.

The percentage of Ab linked to BMP-WT by nonspecific adsorption was almost 20% and could not be ignored. However, the percentages of Ab coupled to magnetosomes by covalent linkage or self-assembly were significantly higher. The linkage rate of ΔF-BMP-FA magnetosomes to Ab was much higher than that of other magnetosomes, indicating that ΔF-BMP-FA is the best candidate for magnetic carrier carriers for IMS (Table 3).

**Table 3:** **Particle size and zeta potential of magnetosomes from various strains**.

**Strain**	**BMP-WT**	**BMP-CA**	**BMP-FA**	**ΔF-BMP-FA**
Hydrated radius (nm)	494.7 ± 18.6	325.2 ± 4.2	334.3 ± 5.1	152.5 ± 0.2
Zeta potential (mV)	−38.27 ± 0.73	−34.09 ± 0.5	−35.12 ± 0.6	−31.09 ± 0.79
Polydispersity	0.354	0.335	0.337	0.230

### Immunomagnetic separation of *V. parahaemolyticus*

Anti-*Vibrio* rabbit polyclonal Ab was linked to recombinant magnetosomes (BMP-CA, BMP-FA, and ΔF-BMP-FA) by self-assembly. WT magnetosomes (BMP-WT) were linked to Ab by BS^3^. Magnetosome-Ab complexes (BMP-A-Ab) were used to capture *V*. *parahaemolyticus*. FQ-PCR was performed with the species-specific gene *tlh* as template. A standard curve was constructed by plotting the threshold cycle (Ct) value against the log of *Vibrio* DNA weight (ng) by linear regression. The number of *Vibrio* cells was estimated based on the amount of DNA attached to the magnetosomes (Table 4).

**Table 4:** **Amount of DNA in Vibrio captured by magnetosome-Ab complexes**.

**Magnetosome**	**BMP-WT**	**BMP-CA**	**BMP-FA**	**ΔF-BMP-FA**
Amount of DNA (ng)	6.092	76.202	70.226	95.581
Number of *Vibrio* (×10^8^)	0.117	1.47	1.35	1.84

Each type of magnetosome (1 mg) was mixed with 1 mL of diluted *V*. *parahaemolyticus* suspension (10^−4^; approximately 2 × 10^8^ cfu). WT magnetosomes bound a much lower number of *Vibrio* than did the other magnetosomes, indicating that many Ab molecules failed to work. ΔF-BMP-FA captured the highest number of *Vibrio*, indicating that ΔF-BMP-FA is the best candidate for a magnetic carrier of IMS for detection of pathogenic *Vibrio*.

## Discussion

Although MTB are ubiquitous and highly abundant in many aquatic habitats, they are very difficult to culture in laboratory or industrial situations. To date, fewer than 20 MTB strains have been successfully isolated and cultured in laboratories worldwide (Greene and Komeili, 2012). *M*. *gryphiswaldense* MSR-1, the type strain of the genus *Magnetospirillum*, is the only MTB strain that has been cultured on a relatively larger scale (in a 1.5-ton fermenter) (Zhang et al., 2011). Mass production of magnetosomes facilitates its detailed biotechnological and nanotechnological studies. These bacterial magnetic nanoparticles are distinguished by unique properties such as ferrimagnetism, nanoscale size, monocrystalline structure, narrow size distribution, uniform morphology, and membrane-bound structure, and thus have been developed and investigated for various potential applications, including immunoassays, magnetofection, therapeutic drug delivery, and enzyme immobilization (Yoshino et al., 2010).

The term “surface display systems” in microbiology refers to a group of powerful techniques that utilize naturally-occurring microbial functional components to express heterologous proteins or peptides. Since the description of the first phage-display system in the mid-1980s, a variety of new systems have been reported for yeast, Gram-positive bacteria, Gram-negative bacteria, bacterial endospores, ribosomes, and mRNAs (Ullman et al., 2011). A recently developed display system based on magnetosomes (BMPs) provides superior performance for immunomagnetic separation, which facilitates high-throughput screening (HTS) (Yoshino et al., 2010). However, only a few studies have investigated or compared the effects of different fusion strategies for MM proteins (Li et al., 2010; Pollithy et al., 2011).

We have recently detected over 200 proteins on magnetosome surfaces (unpubl. data). Some of these proteins are unique to the magnetosome. MamC and MamF, the most abundant proteins on MSR-1 magnetosomes, are small proteins composed respectively of 125 amino acids (12.4 kDa) and 111 amino acids (12.3 kDa). We constructed fusion proteins by fusing *mamC* and *mamF* with the SPA (43 kDa) gene, *spa*. The recombinant magnetosomes were capable of self-assembly with various mammalian Abs without loss of Ab activity. We have evaluated and compared several magnetosome surface display strategies. The highest linkage rate of Abs and recombinant magnetosomes was obtained when the genomic MM genes for constructing fusion proteins were eliminated in recipient strains. It can be explained that there were more functional fusion proteins on the recombinant magnetosomes in the mutant recipient strain than in the wild type MSR-1. The strategy of genetic manipulation developed here would also be applied to the other magnetotactic bacterial pure cultures, such as *M. magneticum* AMB-1 and *Magnetococcus marinus* MC-1. Compared to the chemically produced magnetic beads, it was much more convenient for preparing recombinant magnetosome and antibody complexes because of omitting the coating and linkage procedures. Moreover, the capture efficiency of pathogens and the dispersibility in water were obviously improved comparing with magnetosome—antibody complexes connected with crosslinker (Li et al., 2010). Thus the technique in this study is also suitable for the detection and diagnosis of other pathogens, and makes it simpler, faster, and cheaper.

Our findings are consistent with a previous report that *mamF* deletion in MSR-1 resulted in slightly smaller magnetosome size (Scheffel et al., 2008). Although our fusion proteins contained a fragment corresponding to MamF, the recombinant magnetosomes were smaller than WT magnetosomes. At this stage, we are unclear whether functional MamF is present in the recombinant *M. gryphiswaldense* strain ΔF-FA (which harbors pBBR-mamF-spa), which needs further investigations.

Interestingly, we have detected the proteins MamC and MamF (generally thought to bind to MM) in cytosol and on membranes following fusion with SPA (data not shown). These findings suggest that the binding ability of MM proteins can be affected by fusion with larger soluble proteins, resulting in less SPAs on the surface of recombinant magnetosomes.

Further studies for design of functionalized magnetosomes using the system described here are in progress. Binding abilities of other MM proteins should be investigated in detail. Smaller recombinant proteins containing SPA Ig-binding domains or larger MM binding proteins could also be constructed for more efficient magnetosome display system. The magnetosme surface play technique will include but not limited to improving the separation, detection, and diagnostic analyses, or displaying SPA protein. Other functional moieties, such as enzymes, receptors, peptide hormones, growth factors, autobiotinylation signals, and protein tags for “click chemistry” could be expressed on the magnetosome particles by using our display strategies. These would endow the recombinant magnetosomes with serious functions, or even benefit the formation and reconstruction of magnetic nanostructures, such as magnetic nanotubes and nanowires *in vitro*.

### Conflict of interest statement

The authors declare that the research was conducted in the absence of any commercial or financial relationships that could be construed as a potential conflict of interest.
